# Comparison of glenohumeral joint rotation between asymptomatic subjects and patients with subacromial impingement syndrome using cine-magnetic resonance imaging: a cross-sectional study

**DOI:** 10.1186/s12891-019-2818-3

**Published:** 2019-10-25

**Authors:** Tomonori Kenmoku, Keisuke Matsuki, Nobuyasu Ochiai, Masaru Sonoda, Takumi Ishida, Shuichi Sasaki, Yuji Tanaka, Mitsufumi Nakawaki, Naoshige Nagura, Ryo Tazawa, Yu Sasaki, Scott A. Banks, Masashi Takaso

**Affiliations:** 1 Department of Orthopedic Surgery, School of Medicine, Kitasato University, 1-15-1 Kitasato, Minami-ku, Sagamihara, Kanagawa 252-0570 Japan; 2 Department of Mechanical and Aerospace Engineering, University of Florida, Gainesville, FL USA; 3 Funabashi Orthopaedic Sports Medicine & Joint Center, Funabashi, Japan; 4 Department of Orthopedic Surgery, Chiba University Graduate School of Medicine, Chiba, Japan; 5 Division of Radiology, Seirei Sakura Citizen’s Hospital, Sakura, Japan; 6 Division of Rehabilitation, Kitasato University East Hospital, Sagamihara, Japan; 7 Division of Rehabilitation, Seirei Sakura Citizen’s Hospital, Sakura, Japan; 8 Division of Orthopaedic Surgery, Seirei Sakura Citizen’s Hospital, Sakura, Japan

**Keywords:** Subacromial impingement syndrome, Cine magnetic resonance imaging, Shoulder rotation, Rotator cuff

## Abstract

**Background:**

The purpose of this study to compare glenohumeral joint motion during active shoulder axial rotation between subacromial impingement syndrome (SIS) shoulders and asymptomatic shoulders using cine-magnetic resonance imaging (cine-MRI). Measurement of glenohumeral joint motion via manual intervention does not assess the usual glenohumeral joint motion, and the glenoid surface cannot be confirmed manually. However, cine-MRI can produce clear images of glenohumeral joint rotation. Therefore, we sought to measure the active ROM of the glenohumeral rotation using cine-MRI.

**Methods:**

Seventy-three shoulders in 42 asymptomatic volunteers and 110 SIS shoulders in 103 consecutive patients were included in this study. We evaluated 36 matched pairs (72 shoulders in total) adjusting for baseline characteristics with propensity score matching method. The patients underwent cine-MRI during axial rotation of the adducted arm. During imaging, participants rotated their shoulder from the maximum internal rotation to the maximum external rotation over the first 10 s and then back to the maximum internal rotation over the subsequent 10 s. We assessed internal/external rotation, and compared the asymptomatic and SIS shoulders in this regard. Evaluation of rotation angles was performed on a series of axial images through the humeral head center.

**Results:**

The mean internal rotation angles of the asymptomatic and patient groups were 55° ± 10° and 41° ± 23°, respectively, (*P* = .002; 95% Confidence Interval [CI], 51–58 vs 33–49); the mean external rotation angles were 47° ± 15° and 21° ± 25°, respectively, (*P* < .001; CI, 42–52 vs 13–29).

**Conclusions:**

Compared to asymptomatic shoulders, SIS shoulders showed significantly restricted glenohumeral rotation as determined by cine-MRI. Our results suggested that the significant limitation of active glenohumeral rotation might be associated with rotator cuff dysfunction.

## Background

Subacromial impingement syndrome (SIS) is a common shoulder disorder and is associated with functional impairment and disability of the shoulder [1–5]. The activation of rotator cuff muscles is a fundamental contributor to shoulder joint stability and mobility, which is characterized by centering of the humeral head in the glenoid. Dysfunction of the rotator cuff muscles is considered a major cause of SIS, [1–3] although the precise nature of changes in rotator cuff function in SIS has not been elucidated.

Internal/external glenohumeral rotation is important for shoulder function, because of its association with most shoulder movements, such as abduction or forward flexion [6, 7]. Previous reports have indicated that internal/external glenohumeral rotation is restricted during elevation in shoulders with SIS [8–10]. The humeral head always rotates spontaneously at the beginning of upper extremity elevation, especially from start to 40° [11]. It has been suggested that rotator cuff exercise with the arm by the side improves SIS symptoms [4, 12, 13]. Active axial shoulder rotation requires coordination between the internal and external rotators of the glenohumeral joint. Therefore, patients with SIS, compared to asymptomatic individuals, may have rotational dysfunction of the glenohumeral joint with the arm adducted. To date, however, no study has evaluated internal/external glenohumeral rotation with the arm adducted. The common clinical measurement of the rotational angle is a combination of the angles of scapulothoracic and sternoclavicular motion [14]. Thus, it is difficult to assess glenohumeral joint rotation accurately.

Cine-magnetic resonance imaging (cine-MRI) allows dynamic evaluation of subjects and has been used in various fields, such as dynamic evaluation of cardiac function [15, 16]. Although several analyses of shoulder axial rotation with cine-MRI have been documented, [17–19] the recorded motion was not truly dynamic; images of incremental movement were used instead. Recent advances in MRI systems now allow the acquisition of one to two images per second for cine-MRI, allowing the analysis of dynamic joint motion [20]. Particularly, axial MRI images of the shoulder can provide reliable images for measuring the active rotational angle of the glenohumeral joint noninvasively.

The purpose of this study was to use cine-MRI to compare glenohumeral joint motion during active shoulder axial rotation between SIS shoulders and asymptomatic shoulders. We hypothesized that the range of shoulder axial rotation with the arm in an adducted position in patients with SIS was restricted compared to asymptomatic controls.

## Methods

### Participants

The experimental protocol was conducted in accordance with the guidelines of Kitasato University Medical Ethics Organization for Clinical Research (KMEO B11–87). The Institutional Review Board of our institute approved the protocol for this study, and all participants (or their parents, if they were underage) provided written informed consent.

This study was of a cross-sectional design. Between January 2009 and December 2013, 73 shoulders of 42 asymptomatic volunteers (18 women, 24 men; asymptomatic group) with a mean age of 28 years (range, 21–40) were enrolled in this study (Table 1). The patients had no history of shoulder pain or trauma around the shoulder girdle, including the spine. Because we recruited asymptomatic volunteers at two facilities, two physical therapists in each facility confirmed that the shoulder range of motion (ROM) was not limited and that the painful arc sign, [21] Neer impingement sign, [2] and Hawkins-Kennedy impingement sign [22] were all negative. It was also confirmed via MRI that none of the volunteers had glenohumeral osteoarthritis, rotator cuff tears, and increased signal sign in the glenohumeral joint capsule in the T2-weighted coronal, sagittal, and axial images.

**Table 1:** Demographic data of subjects

	Before			After propensity score matching		
	Asymptomatic group	Patient group	*P*	Asymptomatic group	Patient group	*P*
Shoulders [n]	73	110	–	36	36	–
Age [y]	28 (range, 21–40)	50 (range, 15–81)	< .0001	31 (range, 21–40)	30 (range, 15–54)	.8
Sex	F, 36; M, 37	F, 34; M, 76	.01	F, 9; M, 27	F, 9; M, 27	1.00
Side	D, 41; ND, 32	D, 69; ND, 41	.4	D, 24; ND, 12	D, 28; ND, 8	.4
Flexion [°]	178 ± 3 (CI, 177–178)	159 ± 22 (CI, 155–163)	< .0001	177 ± 3 (CI, 172–177)	163 ± 22 (CI, 155–170)	.0006
Abduction [°]	178 ± 3 (CI, 178–179)	138 ± 39 (CI, 131–146)	< .0001	178 ± 3 (CI, 177–179)	152 ± 36 (CI, 140–164)	.0001
ER [°]	74 ± 9 (CI, 72–76)	68 ± 18 (CI, 65–72)	.2	72 ± 10 (CI, 69–76)	76 ± 16 (CI, 70–82)	.3
IR ^*a*^	T7 (Range, T12-T5)	T11 (Range, B-T5)	< .0001	T7 (Range, T12-T5)	T10 (Range, B-T5)	< .0001

*F* female, *M* male, *D* dominant, *ND* nondominant, *ER* external rotation, *IR* internal rotation, *T* thoracic vertebra, *B* buttock, *CI*, 95% confidence interval

^*a*^IR was determined by the spine level that could be reached by the thumb

Between January 2012 and December 2015, 155 consecutive patients (169 shoulders) with a mean age of 54 years (range, 15–81) suspected of having SIS without global loss of passive ROM (≤ 100° of forward flexion, ≤ 10° of external rotation with the arm adducted, and internal rotation of < the L5 level), [23] underwent cine-MRI at one of our two institutes. Before MRI, we excluded patients who experienced pain at rest or/and both active and passive internal and external rotation with the arm in an adducted position, to eliminate the influence of pain on rotational motion (*n* = 17). However, we included patients who felt pain at the end of rotation (*n* = 40). We also excluded patients with external rotation lag sign, which suggested rotator cuff tears (*n* = 16), [24] and patients with collagen diseases (*n* = 4). A single surgeon confirmed that all patients were positive for one of the following tests: painful arc sign, [21] Neer impingement sign, [2] or Hawkins-Kennedy impingement sign [22]. Fifteen patients (18 shoulders) that were diagnosed with partial or full-thickness rotator cuff tears via MRI (T2-weighted coronal and sagittal images) were excluded. No one showed increased signal or capsule edema around the axillary recess in fat-suppressed T2-weighted MR images, which suggested capsulitis related to glenohumeral joint motion (Sensitivity, 85.3–88.2%; Specificity, 88.2%) [25, 26]. In addition, no one showed severe glenohumeral joint deformity. Thus, 110 shoulders in 103 patients (70 men and 33 women; patient group) with a mean age of 50 years (range, 15–81) were included this study (Table 1).

We noted a significant difference in age and gender when the rotational angles were compared between the asymptomatic subjects and patients with SIS. Hence, we generated 1:1 matched groups to facilitate the comparison between the normal subjects and patients on the basis of the propensity score, which was calculated via multivariate logistic regression analysis for each subject, and included confounders for age, sex, and handedness. After matching the study group by the propensity score, a total of 72 shoulders were considered for this investigation comparing the results of ROM determined by cine-MRI between the asymptomatic subjects and patients (Table 1).

### Clinical assessment

One orthopedic surgeon measured the active ROMs for flexion, abduction, and internal and external rotation of the adducted shoulders with the patients seated. Active ROMs of the shoulder were measured following the concept of Cave and Roberts for defining a zero position of the glenohumeral joint with a goniometer [14]. All patients were also evaluated using the Constant score [27] and the UCLA Shoulder Rating Scale (UCLA scale) [28].

### MRI acquisition

Imaging was performed with a 1.5 T MRI system using a four-channel shoulder array coil (Signa, GE Medical Systems, Milwaukee WI, USA) or with a 1.5 T system with one of the manufacturer’s sized shoulder coils (shoulder 16, Magnetom Aera, Siemens Healthcare, Malvern PA, USA). Cine-MRI of the shoulder was performed using the two-dimensional fast imaging employing steady-state acquisition (FIESTA) technique (GE system) (imaging parameters: TR/TE = 4.6/2.1 msec; flip angle, 20°; bandwidth, ± 62.5 kHz; matrix, 256 × 224; number of excitations, 1.0; field of view, 28 × 28 cm; section thickness, 6.0 mm) or using true fast imaging with steady state precession (True FISP) (Siemens Healthcare) (imaging parameters: TR/TE = 4.91/2.46 msec; flip angle, 20°; bandwidth, 349 Hz/pixel; matrix, 256 × 256; number of excitations, 1.0; field of view, 28 × 28 cm; section thickness, 6.0 mm). Sequential images were recorded at a rate of 1 image/sec during the activity. Image acquisition was performed on axial slices that included the center of the humeral head, which was determined with a best-fit circle for the humeral head on a scout oblique coronal image.

Subjects performed internal and external rotation of the shoulder in the supine position with the arm adducted. Soft plate cushions were placed under the arm to maintain its long axis parallel to the trunk. Acquisition began with the arm fully rotated internally (with the dorsum of the hand on the greater trochanter). They rotated the arm to the maximum external rotation for over 10 s and then reversed to the maximum internal rotation for the subsequent 10 s. Before MRI acquisition, all subjects practiced the motion several times following the instructions of a doctor or therapist. We recorded the motion of at least two sets of the activity for each subject.

### MRI evaluation

We assessed the internal/external rotation angles of the patients, and compared them between the asymptomatic controls and SIS shoulders.

**Fig. 1:**
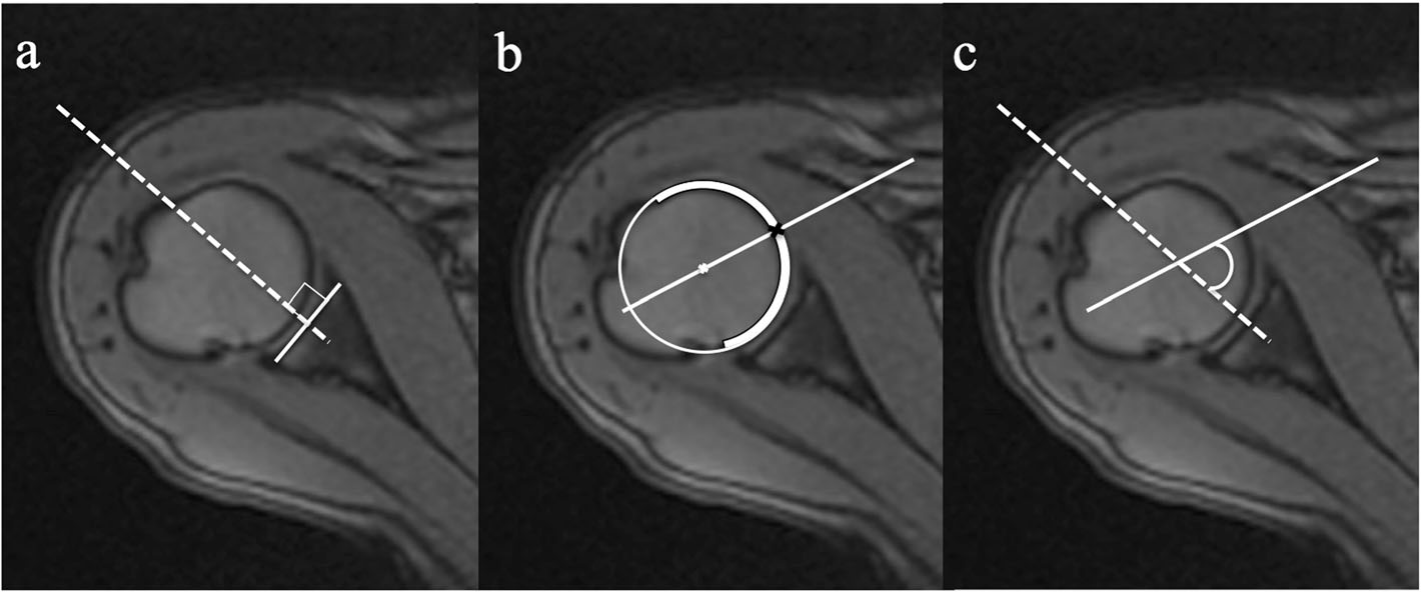
Rotation angle of the glenohumeral joint. The glenoid axis (dotted line) was defined as a line perpendicular to the surface of the glenoid fossa at its midpoint (**a**). The humeral head axis (white line) was defined as a line connecting the midpoint of the articular surface (black **×**) to the center of the humeral head (white dot), which was the center of the best-fit circle (white circle) applied to the humeral head (**b**). The articular surface was identified by the articular cartilage (bold white line). The rotation angle was defined as the angle formed by the glenoid and humeral head axes (**c**)

Rotation angle was defined as the angle formed by the glenoid and humeral head axes (Fig. 1). The glenoid axis was defined as a line perpendicular to the midpoint of the glenoid fossa. The humeral head axis was defined as a line connecting the midpoint of the articular surface and the center of the humeral head that was the center of the best-fit circle applied to the humeral head. When the two axes were parallel, the joint was considered to be in the neutral position. One examiner, who had 10 years’ experience in shoulder research, measured the angle in all subjects. This examiner was blinded to all patient personal and clinical information. Measurement was performed on all recorded activities in each subject, and the largest value was used for further assessment. The maximum angle of internal/external rotation from 2 trials was accepted as the rotational angle in each case.

### Statistical analysis

Statistical analysis was performed using commercial software (JMP Pro version 12.2, SAS Institute Inc., Cary NC, USA). Results are presented as the mean ± standard deviation (SD). The Welch t-test was used to compare quantitative variables. Receiver operating characteristic (ROC) analysis was performed to determine diagnostic cutoff values of rotational angles for SIS. The area under the curve (AUC) was used to assess the accuracy of the analysis, with values > 0.9 considered highly accurate and 0.7–0.9 moderately accurate [29]. We also compared sex and affected side using chi-square test. For all statistical analyses, significance was set at *P* < .05.

Interclass correlation coefficients (ICC) were calculated to investigate inter- and intra-examiner reliability for the angle measurements. For inter-examiner reliability, two shoulder surgeons with 5 and 10 years’ experience in shoulder research, respectively, independently measured 40 randomly selected shoulders, and ICC (2,1) was determined. For intra-examiner reliability, the surgeon with 10 years’ experience measured 40 shoulders twice at a 1-week interval, and ICC (1,1) was determined. All examiners were blinded to the patients’ personal and clinical information.

## Results

### Participants’ demographics

One shoulder surgeon confirmed that no patient had a progressive global restriction of the shoulder that was related to a severe frozen shoulder [23] at least 1 month after MRI. Comparison of the active ROMs of the asymptomatic controls and the SIS shoulders, measured by the goniometer, revealed significant differences in all directions except external rotation (Table 1). The number of patients who were positive in each clinical test is listed in Table 2. The mean Constant score and UCLA scale of patients after propensity score matching were 73.5 ± 13.1 and 19.8 ± 3.4, respectively. The available data of this study included in the Additional file 2.

**Table 2:** The number of patients who were positive in each clinical test

Clinical test	n
The painful arc sign	19
Neer sign	21
Hawkins-Kennedy sign	28
Painful arc sign and Neer sign	0
Painful arc sign and Hawkins-Kennedy sign	4
Neer sign and Hawkins-Kennedy sign	3
All signs	13

Inter- and intra- examiner reliability indicated excellent agreement (ICC [1, 2] = 0.99; 95% Confidence interval [CI], 0.95–0.99; Standard error of mean [SEM] = 1.13; Minimal detectable change [MDC]_95_ = 3.14; ICC [1] = 0.98; CI, 0.97–0.99; SEM = 1.99; MDC_95_ = 7.78).

### Comparison of cine-MRI data between asymptomatic shoulders and shoulders with SIS

**Fig. 2:**
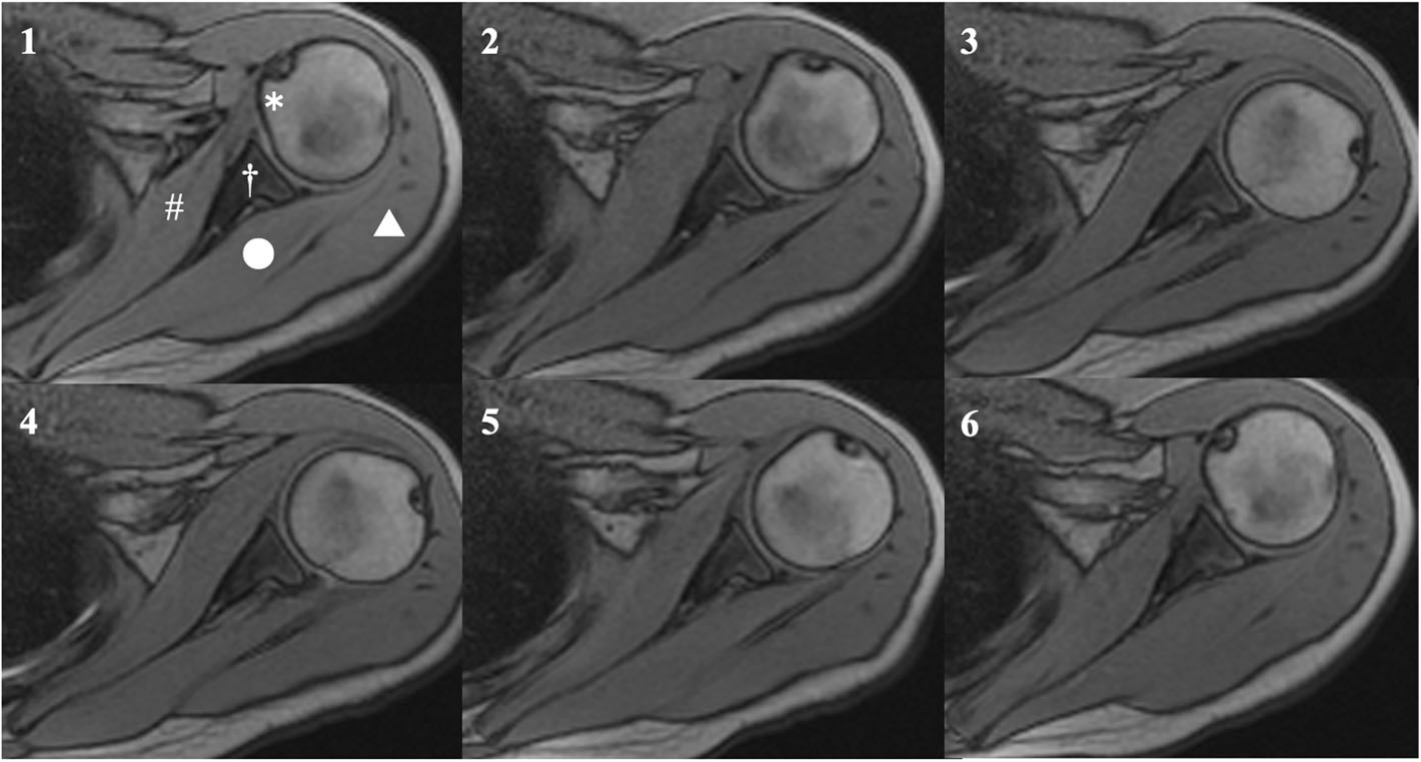
Motion of an asymptomatic volunteer. The left shoulder of a participant. The movement of the subscapularis and infraspinatus muscles of an asymptomatic shoulder was clearly visible using cine-MRI and no blurring was observed. 1, Starting position; 2, Motion in external rotation; 3, Point at the maximum external rotation; 4 and 5, Motion in internal rotation; 6, End position. *, The lesser tuberosity of the humeral head; #, The subscapularis; †, Glenoid; ○, The infraspinatus; △, The deltoid

**Fig. 3:**
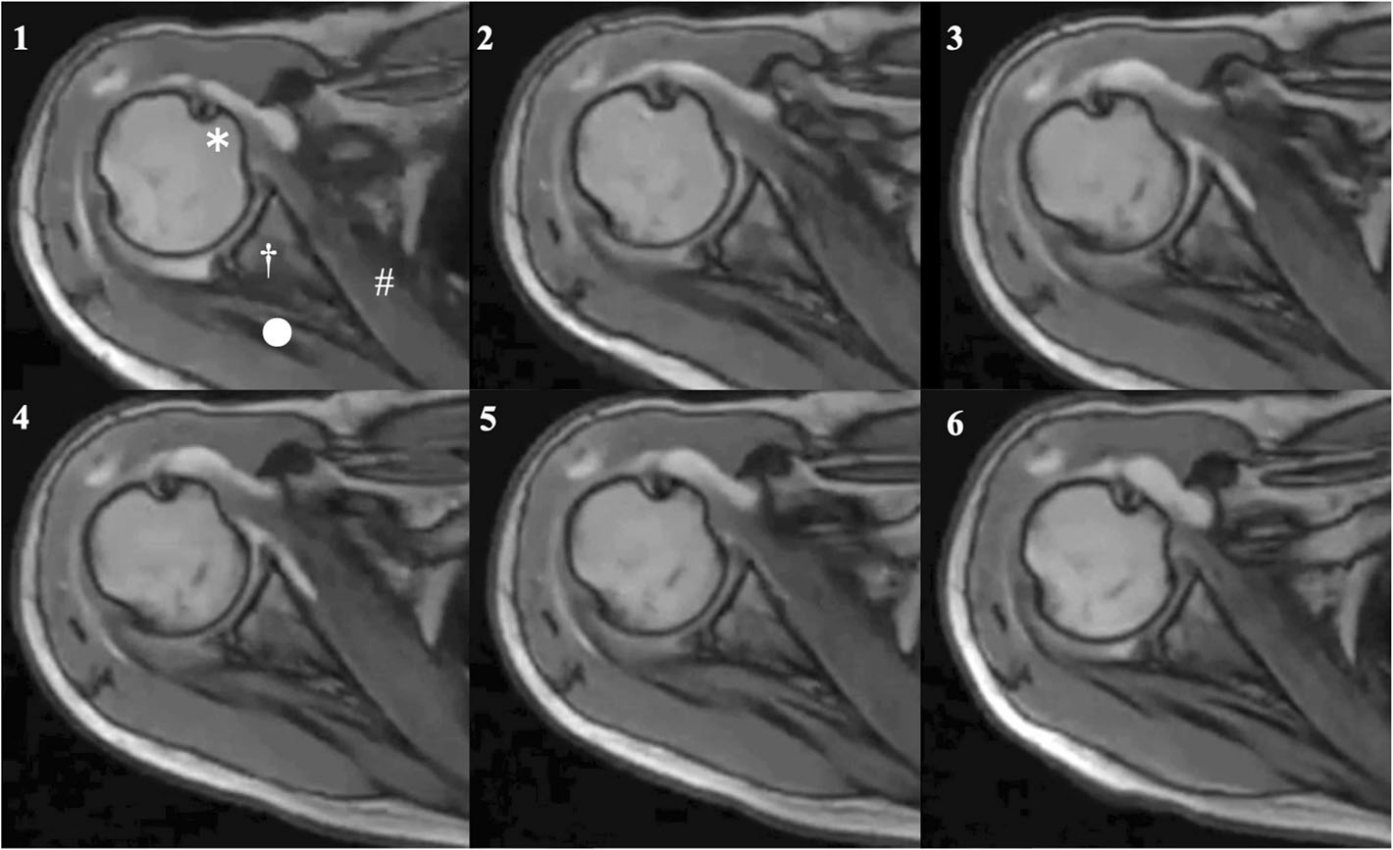
Limitation in external rotation of shoulder with shoulder impingement syndrome. The right shoulder of a patient with shoulder impingement syndrome. Cine-MRI also produced clear images of the movements of the glenohumeral joint rotation in a shoulder with SIS. 1, Starting position; 2, Motion in external rotation; 3, Point at the maximum external rotation; 4 and 5, Motion in internal rotation; 6, End position. *, The lesser tuberosity of the humeral head; #, The subscapularis; †, Glenoid; ○, The infraspinatus

**Fig. 4:**
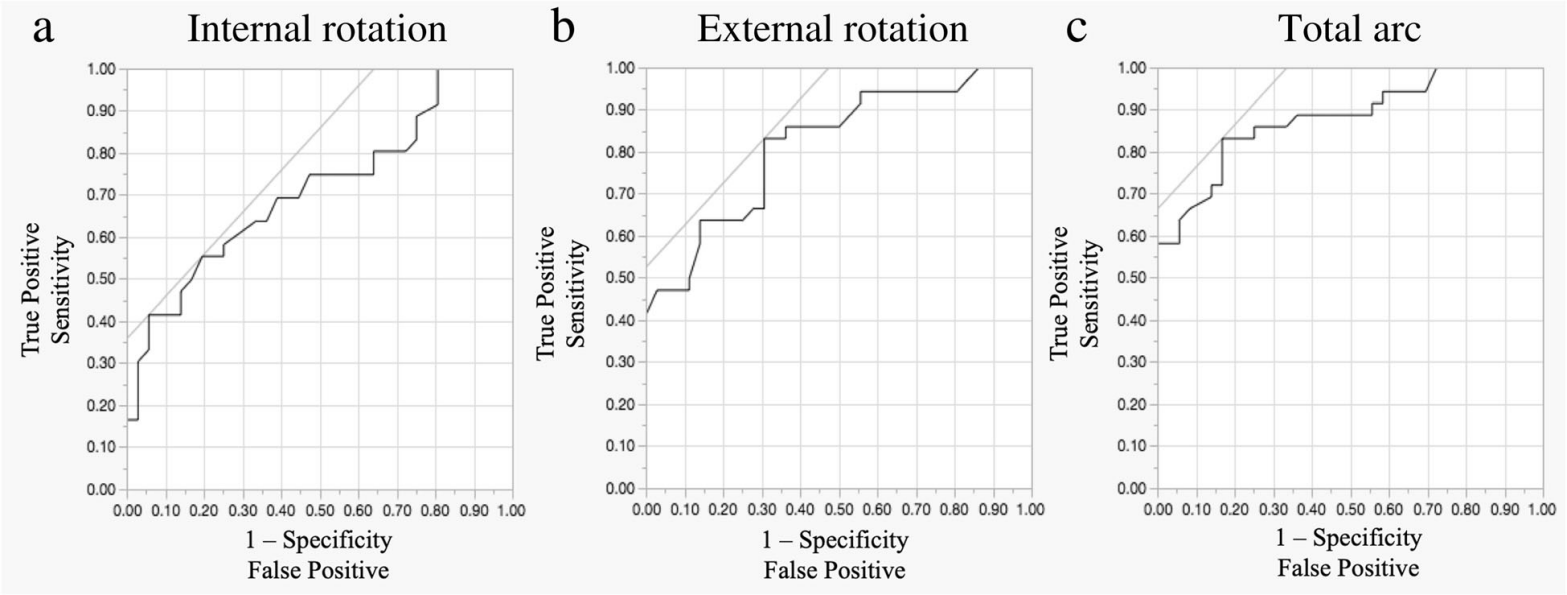
Receiver operating characteristic (ROC) analysis **a**, internal rotation; **b**, external rotation; **c**, total arc. According to ROC analyses, the cutoff values for diagnosis of shoulder impingement syndrome were 47° for internal rotation (sensitivity, 0.56; specificity, 0.81; sensitivity - [1-specificity], 0.36; AUC, 0.71), 41° for external rotation (sensitivity, 0.83; specificity, 0.69; sensitivity - [1-specificity], 0.53; AUC, 0.82), and 86° for total arc (sensitivity, 0.83; specificity, 0.83; sensitivity - [1-specificity], 0.67; AUC, 0.88)


Cine-MRI could obtain scans of the active glenohumeral rotation with vivid clarity in all asymptomatic shoulders and shoulders with SIS (Figs. 2, 3; an additional movie file shows this in more detail (see Additional file 1)). The mean internal rotation angles of the asymptomatic and patient groups were 55° ± 10° and 41° ± 23°, respectively, (*P* = .002; CI, 51–58 and 33–49, respectively; Mean difference [MD], − 14; Standard error difference [SED], 4); mean external rotation angles were 47° ± 15° and 21° ± 25°, respectively, (*P* < .001; CI, 42–52 and 13–29, respectively; MD, 26; SED, 5). The results of ROC analyses showed the cutoff values of rotation angles in cine-MRI studies with a high diagnostic accuracy (Fig. 4).

**Additional file 1.** Active rotation of the left shoulder of a participant (Fig. 2), and the right shoulder of a patient with shoulder impingement syndrome (Fig. 3) captured by cine-MRI. To recreate the coordination of the rotator cuff captured by cine-MRI, we produced a movie with the use of OsiriX software. The process of the image editing was as follows: 1. The files in DICOM format were transferred to a computer with OsiriX software installed. 2. The DICOM files were imported using OsiriX software. 3. After loading all files, the movie export function was enabled to create a Quick Time movie of the 2D volume of DICOM data captured by cine-MRI. The frame rate was set at 1 image/s.


## Discussion

Consistent with our hypothesis, glenohumeral internal/external rotation with the arm adducted was significantly restricted in shoulders with SIS compared to the asymptomatic controls. However, there were no significant difference in the active ROM of external rotation measured physically. Our findings may suggest that rotator cuff exercise with the arm adducted improves SIS symptoms [4, 12, 13].

The significant limitation of active glenohumeral rotation determined by cine-MRI may be associated with rotator cuff dysfunction. The infraspinatus and subscapularis should work as principal external and internal rotators, respectively, during axial rotation of the adducted arm [7, 12, 30, 31]. Imbalance of the rotator cuff muscles is considered one of the causes of SIS, [3, 13, 32] and previous studies have reported that internal/external rotator activities were significantly lower in shoulders with SIS compared to asymptomatic shoulders [33, 34]. Decreased activity of the infraspinatus and subscapularis may have been related to the limited internal/external rotation in SIS shoulders in this study. Additionally, our study sample included individuals with end-range active ROM pain. A bidirectional relationship exists between end-range pain and active ROM (e.g., pain may limit active ROM, and limited active ROM may induce pain); we could not dissociate this relationship. Further studies are required to clarify the contribution of end-range pain to active ROM in SIS.

Many authors have reported that the limitation of glenohumeral rotation is related to symptoms of SIS [8, 9, 35]. The findings of these reports are consistent with our results. The pathology of SIS is considered to predispose the shoulder to mechanical compression in the subacromial space due to excessive superior translation of the humeral head during elevation [2, 3, 36]. The infraspinatus and subscapularis work as depressors of the humeral head during elevation [1, 31]. Therefore, the decreased rotational function of the infraspinatus and subscapularis suggested by our results may also be related to the increased superior translation of the humeral head. Since we did not analyze rotational differences during elevation, further research are necessary to confirm this hypothesis.

Although many shoulders exhibited severe rotational limitation as shown by the MRI measurements, we were unable to detect rotational limitations with the measurement of active ROM of external rotation measured physically. One possible explanation for this discrepancy may be the differences in functional compensation by the body position. Dysfunction of the rotator cuff muscles can be compensated for by the surrounding muscles and scapular motion, and active ROMs are sometimes maintained even in shoulders with cuff muscle palsy [37, 38]. The scapula has a wide range of motion, and its motion can compensate for limitations in glenohumeral rotation in the standing or sitting position [39]. Contrarily, zero point determined by cine-MRI was the point the axes of the humeral head and glenoid were parallel; therefore, we could measure the glenohumeral rotational angle without compensation. In addition, subjects rotated their arm with the elbow extended in the supine position during MRI acquisition in this study. As this position may have restricted scapular motion, we could isolate glenohumeral rotation. Therefore, the difference in external rotational angle between the two methods may be related to whether the compensation around the shoulder should be excluded or not.

Although cine-MRI is diagnostically informative, it is not feasible in most clinical settings and may not provide sufficient resolution. However, diagnostic ultrasound may be a suitable method for quantifying glenohumeral joint angles. Although current ultrasound-based visualization methods are unable to fully quantify rotator cuff coordination, particularly for muscles on the posterior side of the scapula, recent diagnostic and therapeutic advances may make this more feasible in the future [40–42]. Therefore, the cutoff values identified in the present manuscript (Fig. 2) may provide a suitable reference point for diagnostic ultrasound-based assessments of SIS and post-therapeutic gains in the future.

### Limitations

The major limitation of this study was its cross-sectional design. A longitudinal study that would examine changes in rotational angles before and after treatment, to assess relationships between symptoms and the rotational angle, may be required. Second, we did not assess muscle activity in this study. Electromyography (EMG) may be required to confirm decreased function of the rotator cuff muscles; however, it is impossible to record EMG and MRI simultaneously. Therefore, the discussion and conclusions of our results as they relate to alterations in muscle activity are theoretic. Third, we could not completely rule out the potential influences of end-range pain or early stage adhesive capsulitis on glenohumeral rotation, although we excluded patients with pain at rest or/and during rotational motion during the simulation exercise before cine-MRI was performed. Therefore, our findings might also include the influence of a pain-related subliminal limitation during rotation. Fourth, we did not exclude the involvement of systemic diseases that may contribute to abnormal motion, although we eliminated apparent inhibiting factors except rotator cuff dysfunction, such as rotator cuff tears, stiff shoulder, or osteoarthritis. Fifth, we did not include individuals who were unable to perform the necessary shoulder rotation within the gantry of the MRI. Therefore, these findings cannot be generalized to obese individuals with SIS. Sixth, the ROC curves were constructed based on a relatively small sample. Considering that cine-MRI has not been reported in this population, these findings can be used as a reference point for future experimentation with cine-MRI as well as with other emerging imaging methods that may improve the clinical diagnosis of SIS.

## Conclusions

We compared glenohumeral rotational motion between asymptomatic control and SIS shoulders using cine-MRI. Compared to asymptomatic shoulders, SIS shoulders showed significantly restricted glenohumeral rotation. However, no significant differences in active ROMs of external rotation measured with a goniometer were found between the asymptomatic and SIS shoulders. The significant limitation of active glenohumeral joint rotation in this study may be associated with rotator cuff dysfunction.

## Supplementary information

**Additional file 2.** Available data of this study. This excel file included the data of asymptomatic subjects and patients after propensity score matching.

## Data Availability

We have uploaded the dataset we used for this study as an excel file [Available data of propensity].

## Abbreviations

AUC: Area under the curve
CI: Confidence interval
Cine-MRI: Cine-magnetic resonance imaging
ER: External rotation
FIESTA: Fast imaging employing steady-state acquisition
HSD: Honestly significant difference
ICC: Interclass correlation coefficients
IR: Internal rotation
MD: Mean difference
MDC: Minimal detectable change
ROC: Receiver operating characteristic
ROM: Range of motion
SD: Standard deviation
SED: Standard error difference
SEM: Standard error of mean
SIS: Subacromial impingement syndrome
True FISP: True fast imaging with steady state precession
UCLA scale: The University of California, Los Angeles Shoulder Rating Scale
